# Gamma camera imaging characteristics of ^166^Ho and ^99m^Tc used in Selective Internal Radiation Therapy

**DOI:** 10.1186/s40658-024-00633-3

**Published:** 2024-04-06

**Authors:** David Kästner, Anja Braune, Claudia Brogsitter, Robert Freudenberg, Jörg Kotzerke, Enrico Michler

**Affiliations:** 1 Department of Nuclear Medicine, University Hospital Carl Gustav Carus, Technische Universität Dresden, Fetscherstraße 74, 01307 Dresden, Germany; 2 Faculty of Medicine Carl Gustav Carus, Technische Universität Dresden, Dresden, Germany

**Keywords:** SIRT, ^166^Ho, ^99m^Tc, SPECT/CT, Liver, Radioembolization, Spatial resolution, Image quality, Microspheres

## Abstract

**Background:**

The administration of a ^166^Ho scout dose is available as an alternative to ^99m^Tc particles for pre-treatment imaging in Selective Internal Radiation Therapy (SIRT). It has been reported that the ^166^Ho scout dose may be more accurate for the prediction of microsphere distribution and the associated therapy planning. The aim of the current study is to compare the scintigraphic imaging characteristics of both isotopes, considering the objectives of the pre-treatment imaging using clinically geared phantoms.

**Methods:**

Planar and SPECT/CT images were obtained using a NEMA image quality phantom in different phantom setups and another body-shaped phantom with several inserts. The influence of collimator type, count statistics, dead time effects, isotope properties and patient obesity on spatial resolution, contrast recovery and the detectability of small activity accumulations was investigated. Furthermore, the effects of the imaging characteristics on personalized dosimetry are discussed.

**Results:**

The images with ^99m^Tc showed up to 3 mm better spatial resolution, up to two times higher contrast recovery and significantly lower image noise than those with ^166^Ho. The contrast-to-noise ratio was up to five times higher for ^99m^Tc than for ^166^Ho. Only when using ^99m^Tc all activity-filled spheres could be distinguished from the activity-filled background. The measurements mimicking an obese patient resulted in a degraded image quality for both isotopes.

**Conclusions:**

Our measurements demonstrate better scintigraphic imaging properties for ^99m^Tc compared to ^166^Ho in terms of spatial resolution, contrast recovery, image noise, and lesion detectability. While the ^166^Ho scout dose promises better prediction of the microsphere distribution, it is important to consider the inferior imaging characteristics of ^166^Ho, which may affect individualized treatment planning in SIRT.

## Background

Selective Internal Radiation Therapy (SIRT) is a well-established nuclear medicine treatment for primary and secondary liver malignancies. Radioactively labeled microspheres are injected into the hepatic artery and then accumulate predominantly in the tumor tissue. Currently, three different types of microspheres with different physical properties are commercially available, using the high-energy beta-emitters Yttrium-90 (^90^Y) and Holmium-166 (^166^Ho).

Comprehensive treatment planning, including therapy simulation and scintillation camera imaging, is usually performed using Technetium-99m-labeled macroaggregated albumin (^99m^Tc-MAA) or human serum albumin microspheres (^99m^Tc-HSA) as a surrogate for the therapeutic microspheres. The objectives of the pre-treatment workup and scintigraphy are the determination of the liver-lung shunt (LLS) and the detection of abdominal extrahepatic microsphere deposition as well as the prediction of intrahepatic activity distribution as a measure of regional dose. Several studies have shown a limited predictive value of ^99m^Tc-MAA, which may be due to differences in size, shape, and density of the injected particles [1, 2]. The administration of a scout dose of ^166^Ho poly-L-lactic-acid (PLLA) microspheres has recently become available as an alternative to ^99m^Tc particles for SIRT with ^166^Ho. This displays the theoretical advantage since the same microspheres can be used for diagnostics and therapy. It is reported that the ^166^Ho scout dose may be more accurate for the prediction of microsphere distribution and the associated therapy planning [3–5]. However, further investigation regarding the imaging properties of ^166^Ho compared to ^99m^Tc is required with respect to the different physical properties, e.g. gamma energy and emission probability (Table 1). Due to the low gamma emission probability of ^166^Ho (6.7%) and the need for a medium-energy collimator due to the higher energy bremsstrahlung photons, scintigraphic imaging is expected to be inferior to ^99m^Tc in terms of sensitivity, spatial resolution and lesion detectability.

The aim of the current study was to compare the scintigraphic imaging characteristics of both isotopes, considering the objectives of the pre-treatment imaging (liver-lung shunt, extrahepatic deposition, intrahepatic distribution). The influence of collimator type, count statistics, dead time effects, physical properties and patient obesity on spatial resolution, contrast recovery and the detectability of small activity accumulations was investigated. For this purpose, planar and tomographic images of clinically geared phantoms were acquired. In addition, the influence of the imaging characteristics on treatment planning and personalized dosimetry is discussed.

**Table 1:** Physical properties of the isotopes ^166^Ho and ^99m^Tc

	^166^Ho	^99m^Tc
Half-life (h)	26.8	6.0
γ energy (keV)	81 (6.7%) 1379 (0.9%)	141 (89%)
Maximum β^−^ energy (keV)	1770 (48.7%) 1850 (50.0%)	-

## Methods

All measurements were performed on a dual-head Symbia Intevo 6 SPECT/CT system (Siemens Healthineers) with 3/8 inch NaI(Tl) crystals. The rectangular field of view (FOV) was 53.3 cm x 38.7 cm. Data was acquired using a low-energy high-resolution (LEHR) collimator (hole length: 24.05 mm, septal thickness: 0.16 mm, hole diameter: 1.11 mm) for ^99m^Tc and a medium-energy low-penetration (MELP) collimator (hole length: 40.64 mm, septal thickness: 1.14 mm, hole diameter: 2.94 mm) for ^166^Ho and ^99m^Tc to investigate the influence of the collimator. Dual-energy windows were used for both isotopes. For ^99m^Tc, the photopeak window was centered at 140 keV (15% width) with an adjacent lower scatter window at 119 keV (17.6% width). The ^166^Ho photopeak window was set to 81 keV (15% width). An additional scatter window at 118 keV (12% width) was used to correct for down-scattered high-energy photons. Activity for the phantom experiments was measured using a dose calibrator (ISOMED 2010, NuviaTech Healthcare).

### Sensitivity and count rate performance

The sensitivity was measured using a point source placed in the center of the FOV without attenuation and scatter with activities of 146 MBq and 222 MBq for ^99m^Tc and ^166^Ho, respectively. Planar images were acquired for 5 min on a 256 × 256 matrix. The source-detector distance was set to 10 cm.

**Fig. 1:**
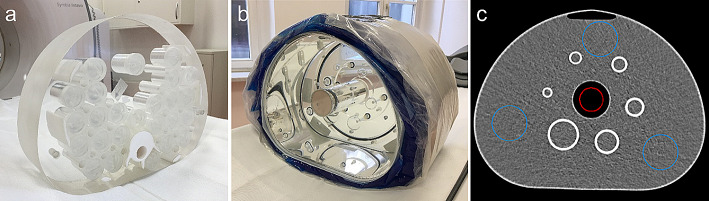
(**a**) Custom-made phantom with a body-shaped cross section, including parts of the ribs and spine with bone-equivalent tissue used for count rate performance measurements. (**b**) NEMA image quality phantom wrapped in cooling gel packs to mimic the attenuation and scattering conditions of an obese patient. The NEMA image quality phantom was used for quantitative evaluation of image quality and investigation of the influence of patient obesity. (**c**) CT image of the NEMA image quality phantom. Transverse slice at the level of the spheres. Background ROIs (blue) and lung insert ROI (red)

The effects of dead time and the resulting deviations of count rate linearity were analyzed depending on the activity at scan time. A custom-made phantom with a body-shaped cross section (diameter 30 cm, width 8 cm), including parts of the ribs and spine with bone-equivalent tissue (Fig. 1a), was used to simulate patient attenuation and scattering conditions. An activity-filled vial was placed in a hole at the center of the phantom. Activities ranging from 190 MBq to 2835 MBq and 154 MBq to 6780 MBq were measured for ^99m^Tc and ^166^Ho, respectively. Planar images were acquired as mentioned above. Anterior and posterior detector-phantom center distances were set to 22 cm and 17 cm, respectively, similar to patient settings. For data analysis, the counts in the photopeak window were plotted against the activity in the phantom. For each isotope and collimator, a data fit was performed according to the paralyzable detector model as follows:

1 $$C\left( A \right) = \alpha \cdot A \cdot {e^{ - \tau \cdot \alpha \cdot A}}$$

where *C* is the measured count rate, *A is* the activity at scan time, *α* describes the linear detector performance and *τ* is the detector dead time. It was specified that the curve passes through the axis origin. In addition, energy spectra were measured and analyzed.

### Quantitative evaluation of image quality

#### Phantom setups

The torso-shaped NEMA image quality (IQ) phantom, containing a fillable background compartment, six fillable coplanar spheres (inner diameter = 10, 13, 17, 22, 28, and 37 mm), and a cylindrical lung insert, was used to evaluate the image quality and spatial resolution. It was filled with three different sphere-to-background activity concentration ratios to resemble clinical setups. To mimic high extrahepatic abdominal deposition, which is characterized by small activity spots with no background activity, the phantom was measured with activity in the six spheres only. Spheres and the background compartment were filled to mimic the activity distribution in the liver with different activity accumulation in the tumor and parenchymal tissue. A sphere-to-background activity concentration ratio of 4:1 was used to mimic liver metastases with moderate uptake, and a sphere-to-background ratio of 8:1 to mimic lesions of hepatocellular carcinoma (HCC) with higher activity uptake relative to the parenchyma. The activity concentrations in the spheres and background were chosen to be similar to those in clinical practice, assuming a patient with a liver volume of 2000 ml containing 150 MBq of ^99m^Tc or 250 MBq of ^166^Ho, which are standard activity values for pre-treatment SIRT diagnostics. A tumor fraction of 10% (200 ml) and a homogeneous activity distribution in tumors and liver parenchyma were assumed. The actual activity concentrations at the time of imaging are shown in Table 2. The phantom was also measured after being wrapped in cooling gel packs (Fig. 1b) to mimic the attenuation and scattering conditions of an obese patient. The cooling packs form an additional thickness of approximately 3 cm around the phantom. The attenuation coefficient of the cooling packs was similar to that of water.

**Table 2:** Information on the measurements with the NEMA image quality phantom filled with ^99m^Tc and ^166^Ho in different phantom setups (spheres without background; sphere-to-background activity concentration ratio 8:1 and 4:1)

Nuclide	Phantom setup	Activity concentration in spheres (kBq/ml)	Activity concentration in background (kBq/ml)	Sphere-to-background activity concentration ratio
^99m^Tc	spheres without background	698.0	-	-
	8:1 contrast (performed twice)	338.8 419.6	39.6 44.6	8.56:1 7.86:1
	4:1 contrast	213.7	52.4	4.08:1
^166^Ho	spheres without background	1376.0	-	-
	8:1 contrast (performed twice)	719.2 589.5	85.7 74.4	8.39:1 7.92:1
	4:1 contrast	386.6	91.5	4.23:1

#### Planar imaging of the NEMA image quality phantom

For the evaluation of the planar scintigraphic imaging characteristics of ^99m^Tc and ^166^Ho, planar anterior and posterior images were acquired for each phantom setup with an acquisition time of 5 min and an image matrix of 256 × 256. Additional measurements of ^99m^Tc with the MELP collimator and a reduced acquisition time of 22 s were performed to investigate the influence of the used collimator and the low gamma emission probability of ^166^Ho compared to ^99m^Tc. The reduced acquisition time was chosen based on the ratio of the gamma emission probabilities of ^166^Ho to ^99m^Tc. Planar scatter correction was applied for ^166^Ho. The images were evaluated with regard to the pre-treatment determination of the liver-lung shunt in SIRT, which is usually based on planar scans. A region of interest (ROI) was placed in the phantom simulating the liver and another ROI was placed above the phantom in an area of no activity. The ratio of the counts in the ROI outside the phantom to the sum of the counts in both ROIs was calculated as a measure of apparent lung shunt.

#### SPECT/CT acquisition and reconstruction parameters

All SPECT data was acquired with 120 projections (60 per head, 20 s per projection) over a non-circular 360° orbit using step-and-shoot mode, followed by a low-dose CT scan (130 kV, 20 mAs, 2.5 mm slice thickness) for attenuation correction. The images were reconstructed to a voxel size of 2.4 × 2.4 × 2.4 mm^3^ using the 3D OSEM algorithm (Flash 3D; Siemens Healthineers) with 8 iterations and 8 subsets for ^99m^Tc and 16 iterations and 8 subsets for ^166^Ho according to our clinical routine. Gaussian post-reconstruction filtering of 9 mm was applied. Scatter correction for ^99m^Tc was performed using the dual-energy window method with an adjacent lower scatter window and a scatter fraction k-factor of 0.5. For ^166^Ho the photopeak was corrected for scatter with the upper scatter window at 118 keV and a k-factor of 1.4, as previously investigated [6]. Additional measurements of ^99m^Tc with the MELP collimator and a reduced scan time of 1.5 s per projection were performed to investigate the influence of the used collimator and the low gamma emission probability of ^166^Ho compared to ^99m^Tc. The acquisition times were adjusted over time according to the respective half-life to obtain comparable count statistics. To obtain information on statistical variation and reproducibility of the results, measurements of the NEMA IQ phantom at 8:1 contrast and standard phantom setup were performed exemplarily twice. The average and standard deviation of both measurements were used for further analysis.

#### Influence of count statistics

The influence of count statistics on SPECT image quality was evaluated by examining the reconstructed images of the NEMA IQ phantom with a sphere-to-background ratio of 8:1 for different acquisition times. SPECT images with projection times of 5 s, 10 s, 15 s, and 20 s for ^99m^Tc and 10 s, 20 s, and 30 s for ^166^Ho were analyzed.

#### Image analysis

The SPECT images were evaluated in terms of contrast recovery, image noise, detectability, relative count error in the lung insert, and spatial resolution. The six hot spheres were segmented using 3D isocontours with a 50% background-adapted threshold for each sphere according to [7]. Three cylindrical volumes of interest (VOI) (45 mm diameter, 150 mm length) were defined in the phantom background (Fig. 1c). The lung insert was delineated with a cylindrical centered VOI (30 mm diameter, 130 mm length) similar to the NEMA NU 2-2018 protocol [8].

The contrast recovery coefficient (CRC) was calculated for each of the six hot spheres as follows:

2 $$CRC = \frac{{\frac{{{{\mathop N\limits^ - }_{\text{S}}}}}{{{{\mathop N\limits^ - }_{{\text{BG}}}}}} - 1}}{{R - 1}} \cdot 100\%$$

where ${\stackrel{-}{N}}_{\text{S}}$ is the mean number of counts in the sphere VOI, ${\stackrel{-}{N}}_{\text{BG}}$ is the mean number of counts in the background VOIs, and $R$ is the true sphere-to-background activity concentration ratio. The relative count error in the lung insert ($\varDelta {N}_{\text{lung}})$ was determined as:

3 $$\Delta {N_{{\text{lung}}}} = \frac{{{{\mathop N\limits^ - }_{{\text{lung}}}}}}{{{{\mathop N\limits^ - }_{{\text{BG}}}}}} \cdot 100\%$$

where ${\stackrel{-}{N}}_{\text{lung}}$ is the mean number of counts in the lung insert VOI. To evaluate the image noise, the noise coefficient of variation (${CV}_{\text{B}\text{G}}$) was calculated using:

4 $$C{V_{{\text{BG}}}} = \frac{{{\sigma _{{\text{BG}}}}}}{{{{\mathop N\limits^ - }_{{\text{BG}}}}}} \cdot 100\%$$

where ${\sigma }_{\text{BG}}$ is the standard deviation of all voxels within the three background VOIs. The contrast-to-noise ratio (CNR) was used to asses object detectability. It was calculated for each sphere as follows:

5 $$CNR=\frac{{\stackrel{-}{N}}_{\text{S}}-{\stackrel{-}{N}}_{\text{BG}}}{{\sigma }_{\text{BG}}}$$

The tomographic spatial resolution was determined based on the analysis of radial profiles through the homogeneously filled phantom spheres in the reconstructed images according to [9]. The full width at half maximum (FWHM) of the point spread function was assessed using the software Rover (version 3.0.60h, ABX, Germany).

## Results

### Sensitivity and count rate performance

The sensitivities measured for the photopeak window for ^99m^Tc were 93.1 cps/MBq and 125.3 cps/MBq for the LEHR and MELP collimator, respectively. For ^166^Ho the sensitivity was much lower at 16.4 cps/MBq for the MELP collimator.

**Fig. 2:**
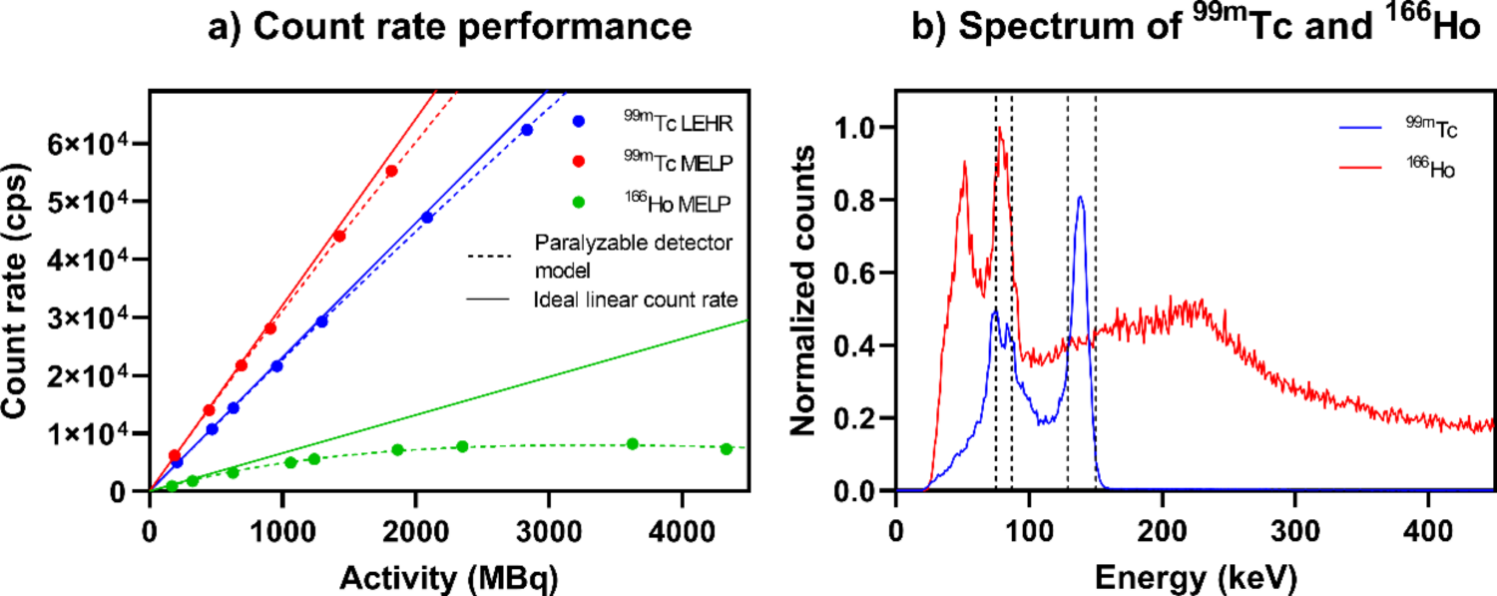
(**a**) Count rate performance measured for ^99m^Tc with LEHR and MELP collimator and for ^166^Ho measured with MELP collimator. (**b**) ^99m^Tc and ^166^Ho spectrum obtained in phantom setup used for count rate performance. The curves are normalized to the sum of the counts in each photopeak window. The dashed lines represent the ^99m^Tc photopeak window centered at 140 keV and the ^166^Ho photopeak window at 81 keV

Figure 2a shows the count rate response for ^99m^Tc measured with the LEHR and MELP collimator and for ^166^Ho measured with the MELP collimator. With increasing activity, there was an increasing deviation from the ideal linear count rate response. The count loss was higher for ^166^Ho than for ^99m^Tc. The activity leading to a 5% count loss was 169 MBq for ^166^Ho with MELP collimator and 3141 MBq and 1611 MBq for ^99m^Tc with LEHR and MELP collimator, respectively. In Fig. 2b, the measured energy spectra of ^99m^Tc and ^166^Ho normalized to the sum of the counts in each photopeak window are shown. It illustrates the photopeak of ^99m^Tc at 141 keV and the characteristic K_α_ and K_β_ radiation of lead at approximately 74 keV and 85 keV. In the energy spectrum of ^166^Ho, the photopeak at 81 keV, the characteristic X-ray radiation of ^166^Ho at 49 keV and a pronounced bremsstrahlung continuum above the photopeak window can be seen. For the equal number of counts in the photopeak window, approximately six times more counts were detected for ^166^Ho than for ^99m^Tc over the entire detector energy range.

### Quantitative evaluation of image quality

#### Planar imaging of the NEMA image quality phantom

**Fig. 3:**
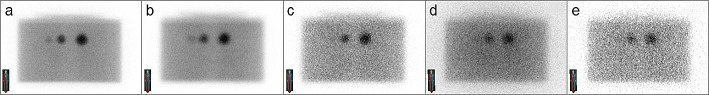
Planar posterior gamma camera images of the NEMA image quality phantom at a sphere-to-background activity concentration ratio of 8:1. (**a**) ^99m^Tc with LEHR collimator, (**b**) ^99m^Tc with MELP collimator, (**c**) ^99m^Tc with MELP collimator and reduced scan time, (**d**) ^166^Ho with MELP collimator, (**e**) ^166^Ho with MELP collimator and planar scatter correction

Figure 3 shows the NEMA IQ phantom planar images of ^99m^Tc and ^166^Ho with a sphere-to-background ratio of 8:1. The ^99m^Tc images featured a better detectability of the spheres and a lower image noise compared to ^166^Ho. The uncorrected image with ^166^Ho showed many artificial counts in the activity-free background, whereas the scatter-corrected image showed reduced number of counts in the activity-free background.

The calculated apparent lung shunt was 2.0% for the ^99m^Tc scans with the LEHR collimator and 1.9% and 1.7% for the ^99m^Tc scans with the MELP collimator with standard and reduced scan time, respectively. For ^166^Ho, the apparent lung shunt was 13.0% for the scans without scatter correction and was reduced to 2.3% with planar scatter correction.

#### Influence of count statistics

**Fig. 4:**
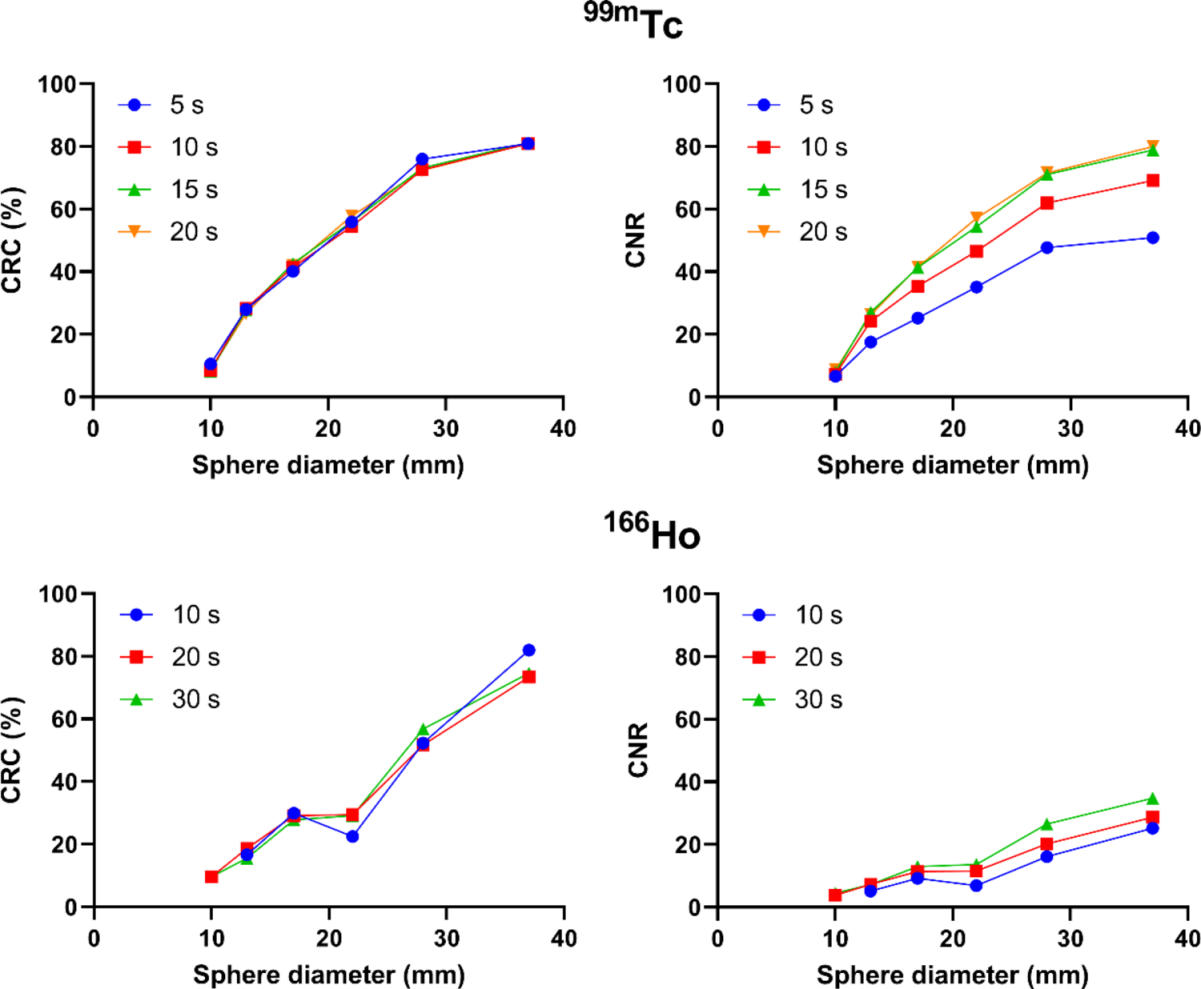
Contrast recovery coefficients (%) and contrast-to-noise ratios as a function of sphere diameter for ^99m^Tc (upper row) and ^166^Ho (lower row) for different projection times. Data was determined using the NEMA image quality phantom at a sphere-to-background activity concentration ratio of 8:1

In Fig. 4, contrast recovery coefficients and contrast-to-noise ratios are shown as a function of sphere diameter for measurements with different acquisition times. For both isotopes, the CRC was almost identical for measurements with different projection times and decreased continuously with decreasing sphere diameter. Spatial resolution, image noise, and lung count error for the different acquisition times are listed in Table 3. The background noise level decreased with increasing acquisition time for ^99m^Tc and ^166^Ho, but was more than twice as high for ^166^Ho compared to ^99m^Tc. For ^99m^Tc, there was no visible improvement in the noise level for the measurement with 20 s projection time compared to the measurement with 15 s projection time. The spatial resolution was comparable between the images of different acquisition times and was approximately 12.5 mm for ^99m^Tc and 15.2 mm for ^166^Ho. For the measurement with ^166^Ho and an acquisition time of 10 s per projection, the smallest sphere (d = 10 mm) could not be evaluated due to the high image noise in the background.

**Table 3:** Spatial resolution (FWHM), image noise (CV_BG_), and lung count error (ΔN_Lung_) determined using the NEMA image quality phantom filled with ^99m^Tc and ^166^Ho at 8:1 contrast with different scan times

Phantom setup	scan time per projection	FWHM (mm)	CV_BG_ (%)	ΔN_Lung_ (%)
^99m^Tc, 8:1 contrast	5 s	12.8	12.0	22.9
	10 s	12.5	8.9	22.5
	15 s	12.3	7.8	22.5
	20 s	12.3	7.7	22.3
^166^Ho, 8:1 contrast	10 s	15.4	24.0	33.3
	20 s	15.1	18.9	31.9
	30 s	15.2	15.2	29.6

#### Influence of collimator type

Table 4 shows the spatial resolution, image noise, and lung count error for ^99m^Tc measurements with LEHR and MELP collimator for the different phantom setups. The measurements with the MELP collimator showed a slightly higher count error in the lung insert than the measurements with the LEHR collimator. Image noise was slightly lower with the MELP collimator than with the LEHR collimator at 8:1 contrast, and identical for both collimators at 4:1 contrast, with a noise level of 7.2%. The spatial resolution was approximately 2 mm worse when using the MELP collimator.

**Table 4:** Spatial resolution (FWHM), image noise (CV_BG_), and lung count error (ΔN_Lung_) determined using the NEMA image quality phantom filled with ^99m^Tc and ^166^Ho in different phantom setups (spheres without background, contrast 8:1 and 4:1, normal and obese phantom setup) with standard and adapted acquisition parameters (collimator type, reduced projection time). For measurements performed twice, results are presented as the mean and standard deviation

Phantom setup		FWHM (mm)	CV_BG_ (%)	ΔN_Lung_ (%)
8:1 contrast	^99m^Tc	12.2 ± 0.1	7.6 ± 0.1	23.5 ± 1.2
	^99m^Tc obese	12.6	8.4	26.9
	^99m^Tc MELP	14.3 ± 0.2	6.8 ± 0.3	26.8 ± 1.4
	^99m^Tc MELP 1.5 s	14.3	15.8	23.8
	^166^Ho	15.2 ± 0.1	18.0 ± 0.9	30.9 ± 1.1
	^166^Ho obese	15.6	22.6	33.3
4:1 contrast	^99m^Tc	12.7	7.2	23.1
	^99m^Tc MELP	15.4	7.2	24.6
	^99m^Tc MELP 1.5 s	15.3	14.5	23.6
	^166^Ho	16.0	17.7	31.8
Spheres without background	^99m^Tc	10.1	-	-
	^99m^Tc MELP	12.0	-	-
	^99m^Tc MELP 1.5 s	12.2	-	-
	^166^Ho	12.5	-	-

#### Comparison of the imaging of ^99m^Tc and ^166^Ho with different phantom setups

**Fig. 5:**
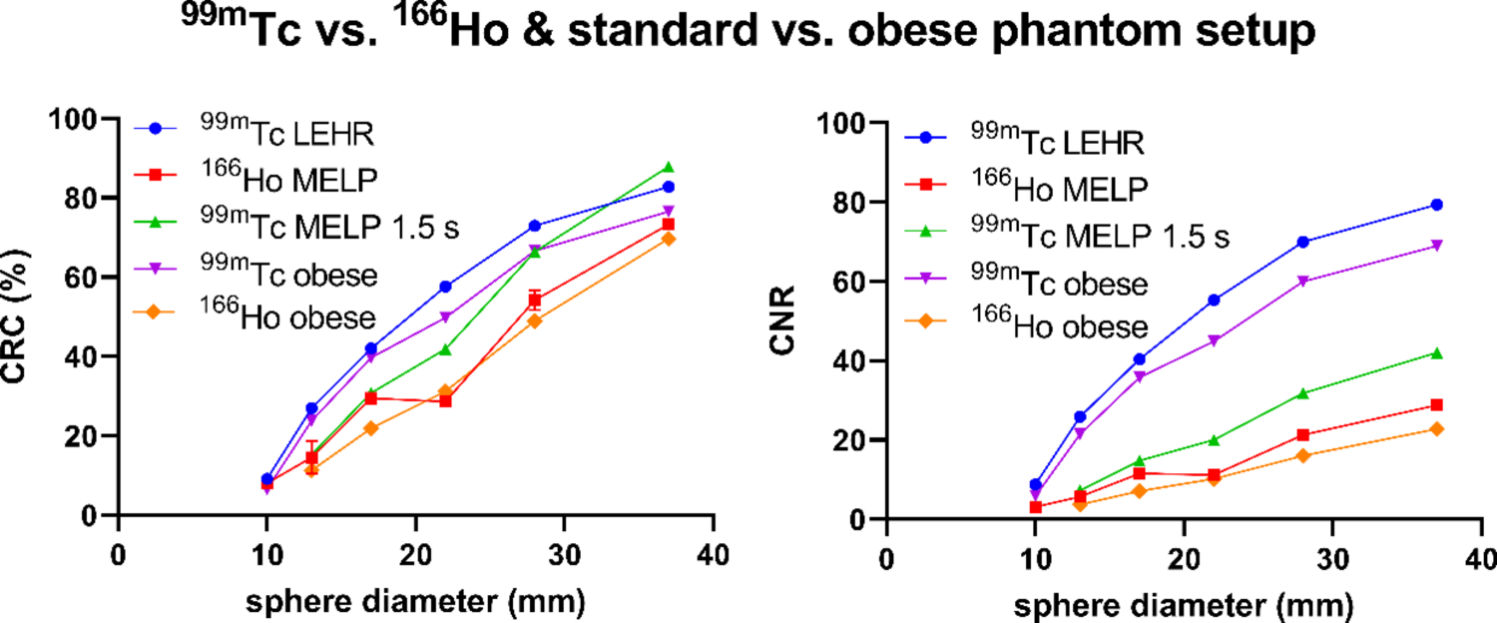
Contrast recovery coefficients (%) and contrast-to-noise ratios as a function of sphere diameter determined using the NEMA image quality phantom at a sphere-to-background activity concentration ratio of 8:1. ^99m^Tc acquired with LEHR collimator and with MELP collimator and reduced scan time (1.5 s projection time) and ^166^Ho acquired with MELP collimator. ^99m^Tc and ^166^Ho acquired with the modified phantom setup mimicking patient obesity. For measurements performed twice, results are presented as the mean and standard deviation (error bars)

Figure 5 shows the CRC and the CNR as a function of sphere diameter for the measurements using the NEMA IQ phantom with 8:1 contrast of ^99m^Tc and ^166^Ho with standard acquisition parameters and for ^99m^Tc with MELP collimator and reduced scan time. For the measurements with ^99m^Tc, the CNR was higher for all spheres compared to ^166^Ho. The CNRs of the largest sphere was 79.4 and 42.1 for ^99m^Tc with standard and adjusted parameters, respectively, and 28.8 for ^166^Ho. The CRC decreased continuously with decreasing sphere diameter for ^99m^Tc. This was similar for ^166^Ho, except that the CRC of the 17 mm and the 22 mm sphere was similar. The smallest sphere (d = 10 mm) could not be evaluated for the measurement with ^99m^Tc and adapted parameters due to the high noise level. Spatial resolution, image noise, and lung count error for the different phantom setups are listed in Table 4. The spatial resolution for the ^99m^Tc images with standard parameters was up to 3 mm better than for ^166^Ho. The spatial resolution was better in the images of the phantom with no background activity and a sphere-to-background ratio of 8:1 compared to 4:1. The image noise for the ^99m^Tc measurement with adjusted parameters was worse than the ^99m^Tc standard (noise level 15.8% vs. 7.6%), but better than ^166^Ho with a noise level of 18.0%. The results of the measurements performed twice showed only slight deviations, which are mostly not visible in the CRC and CNR figures (Fig. 5).

**Fig. 6:**
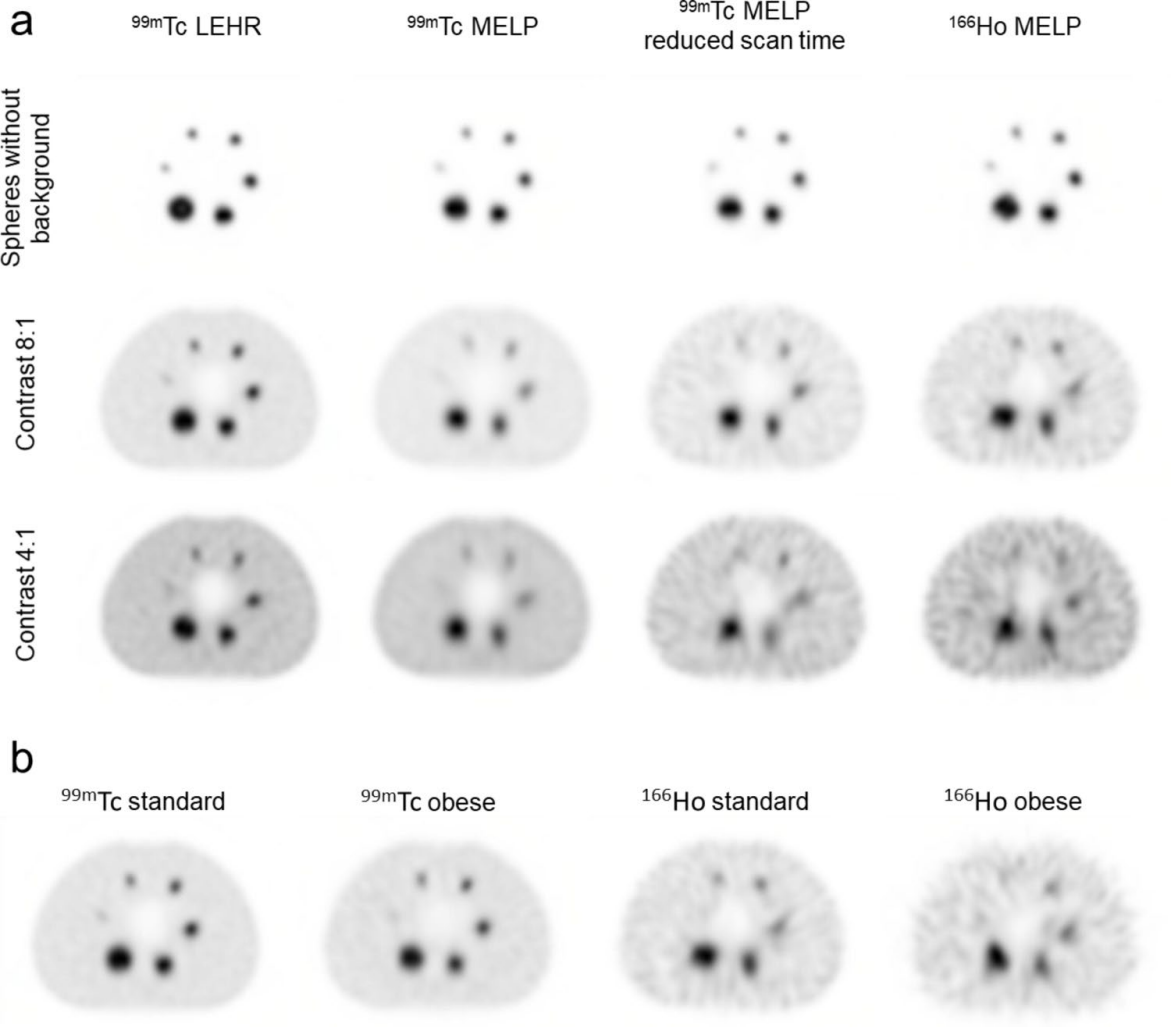
(**a**) SPECT images of the NEMA image quality phantom filled with ^99m^Tc and ^166^Ho in different phantom setups (spheres without background activity, sphere-to-background activity concentration ratio 8:1 & 4:1). For ^99m^Tc, images were acquired with LEHR collimator, MELP collimator and reduced scan time. ^166^Ho images were acquired using the MELP collimator. Transverse slices at the level of the spheres are shown. (**b**) SPECT images of the NEMA image quality phantom filled with ^99m^Tc and ^166^Ho at a sphere-to-background activity concentration ratio of 8:1, acquired with the standard phantom setup and the modified phantom mimicking patient obesity. Transverse slices at the level of the spheres are shown

Figure 6a shows the reconstructed transverse images of all NEMA IQ phantom setups of ^99m^Tc and ^166^Ho with standard acquisition parameters and for ^99m^Tc with MELP collimator and reduced scan time. For the measurement with activity in the spheres only, all spheres were visible for both isotopes. When comparing the 8:1 contrast images, for ^166^Ho and ^99m^Tc measured with the MELP collimator, the smallest sphere (d = 10 mm) was not detectable and the high background noise level is evident. In the 4:1 contrast images, the smallest sphere was not detectable for ^99m^Tc and only the three largest spheres (d ≥ 22 mm) were detectable for ^166^Ho. The images with activity in the background showed a distortion of the spheres for the measurements with ^99m^Tc with reduced scan time and for ^166^Ho.

#### Influence of an obese body

The use of the obese phantom setup resulted in an increase in the lung count error and the image noise and a slight decrease in the CRC for both isotopes (Table 4). This has resulted in a decrease in the CNR (Fig. 5) and therefore a decrease in the detectability of the hot spheres. For the measurement with ^166^Ho in the obese phantom setup, the smallest sphere (d = 10 mm) could not be evaluated due to the high noise level. The spatial resolution was slightly worse when using the obese phantom setup (^99m^Tc: 12.6 mm; ^166^Ho: 15.6 mm) compared to the standard phantom setup (^99m^Tc: 12.2 mm; ^166^Ho: 15.2 mm). The reconstructed transverse images of the NEMA IQ phantom measurements of ^99m^Tc and ^166^Ho with standard and obese phantom setup are shown in Fig. 6b. The measurement of ^166^Ho with the obese phantom setup showed a significantly worse detectability of the spheres and a blurring at the edge of the phantom compared to the standard phantom setup.

## Discussion

The scintigraphic imaging properties of ^99m^Tc and ^166^Ho have been well studied and characterized [10–13], and there also is a comparison of the three isotopes (^99m^Tc, ^90^Y, and ^166^Ho) used in radioembolization [14]. However, these studies are primarily oriented towards the NEMA performance measurement guidelines and do not consider the clinical imaging aspects of SIRT. Our study focused on the scintigraphic imaging characterization of ^99m^Tc and ^166^Ho using phantom geometries, considering the aspects of pre-treatment imaging in radioembolization. The influence of the collimator type, count statistics, dead time effects isotope properties and patient obesity on image quality (spatial resolution, contrast recovery, image noise, lesion detectability) was evaluated in detail.

As expected, the sensitivity for ^166^Ho was significantly lower than for ^99m^Tc, but higher than the expected sensitivity according to the low gamma emission probability of ^166^Ho compared to ^99m^Tc. This is due to the presence of lead X-rays and bremsstrahlung photons in the 81 keV photopeak window. The MELP collimator showed a higher planar sensitivity than the LEHR collimator for ^99m^Tc due to the larger hole diameter and the resulting larger acceptance angle. The count rate performance of ^99m^Tc showed an almost linear detector response and there is no significant count loss for the clinically used activity range. The activity resulting in a 2% count loss was 1237 MBq for ^99m^Tc with LEHR collimator. This is in a similar order of magnitude as the activity values of about 1 GBq reported by Ryu et al. [12] and Elschot et al. [14]. The progressive count loss for ^166^Ho with increasing activity is caused by dead time effects due to the high sum of counts from detected bremsstrahlung photons over the entire energy range of the detector. In this study, a value of 736 MBq was found, leading to a count rate loss of 20% for ^166^Ho. The work of Stella et al. [15] reported similar count loss values. For a ^166^Ho scout dose of 250 MBq, count loss can be neglected, but at high activity levels measured after radioembolization, dead time effects must be considered, especially for quantitative SPECT imaging. Sensitivity and count rate performance are directly related to count statistics and image noise. Analyses of the effect of count statistics showed a continuous decrease in image noise with increasing count statistics. Due to the low gamma emission probability of ^166^Ho, the noise level is significantly higher than for ^99m^Tc. It was found that the count statistics did not have a significant effect on spatial resolution and CRC. However, image noise, CNR, and therefore lesion detectability decrease with lower count statistics. Except for the measurements of ^99m^Tc with 15 s and 20 s projection time, where similar values were found for image noise and CNR. Therefore, these results may be useful for clinical routine, as the 15 s projection time images provide an equivalent image quality. The use of the MELP collimator instead of the LEHR collimator degraded the resolution by approximately 2 mm FHWM for ^99m^Tc. This is a consequence of the wider acceptance angle of the MELP collimator and the larger source to detector (crystal surface) distance due to the thicker MELP collimator. The use of the MELP collimator for ^99m^Tc was not considered for clinical routine, but only for investigation of the collimator influence on image quality.

The qualitative and quantitative evaluation of image quality revealed that planar and SPECT imaging was worse for ^166^Ho compared to ^99m^Tc regarding spatial resolution, contrast recovery, image noise, lesion detectability, and lung count error (Figs. 3, 5 and 6; Table 4). To investigate the influence of the properties of the two isotopes on image quality, measurements with ^99m^Tc were performed using the MELP collimator and reduced acquisition time to achieve similar count statistics. These measurements also showed better image characteristics compared to ^166^Ho, demonstrating that additional aspects affect the inferior image quality of ^166^Ho. The lower gamma energy of ^166^Ho (81 keV) compared to ^99m^Tc (141 keV) leads to a decrease of the spatial resolution and a noticeably greater blurring. The effect of gamma energy on gamma camera spatial resolution was previously investigated by Holstensson et al. [16]. The high energy bremsstrahlung photons of ^166^Ho lead to septal penetration and crystal scatter and they produce lead X-rays in the collimator which are detected in the 81 keV energy window. These photons have a loss of spatial information and result in a worse image quality regarding image noise and contrast recovery. There is a high percentage of scatter when measuring ^166^Ho, leading to a high lung count error and the detection of scattered photons in compartments with no activity. The high noise level in the images with ^99m^Tc with reduced scan time and with ^166^Ho results in a distortion of the spheres as they merge with the noisy background [17]. For both isotopes, the CRC decreased continuously with decreasing sphere diameter due to the partial volume effect (Fig. 5). For ^166^Ho, the 22 mm sphere unexpectedly had a similar CRC value as the 17 mm sphere. This could be due to the sphere position in the phantom and the larger detector radius in the lateral position when using auto-contouring [18]. For the ^166^Ho measurement with the obese body phantom setup the CRC of the 22 mm sphere was similar to the CRC of the standard phantom setup measurement, but the CRC of the 17 mm sphere was lower. This is most likely caused by the larger detector radius in the anterior position while the lateral radius is the same due to the patient couch. The exemplary twice-performed measurements show a low statistical variation. This indicates that the results are reproducible and are not coincidental findings.

The measurements with the NEMA IQ phantom mimicking an obese patient resulted in a degraded image quality in terms of CRC, CNR, image noise, lung count error, and spatial resolution for both isotopes. This is a result of the additional material around the phantom leading to increased photon attenuation and scattering. In addition, the source-detector-distance is larger due to the additional volume, which may explain the worse spatial resolution [19]. The reduced CNR indicates that the detectability of small lesions is more challenging in obese patients. Reduced lesion detectability in obese patients is already reported by Lin et al. [20] for ^99m^Tc planar imaging. Peters et al. [21] previously investigated the influence of an obese body on SPECT imaging with ^99m^Tc using different phantoms and reported slightly reduced recovery coefficients similar to our results. Degraded image quality for an obese phantom setup was also reported for PET imaging by Braune et al. [22]. The reduction in image quality for an obese body was greater for ^166^Ho than for ^99m^Tc due to the lower gamma energy and therefore greater attenuation in the material. The half-value layers for ^99m^Tc and ^166^Ho in water are 4.59 mm and 3.96 mm, respectively [23].

The different imaging characteristics can have an impact on pre-treatment diagnostic and treatment planning in SIRT. The planar phantom images showed a high apparent lung shunt for ^166^Ho due to the high number of scattered photons. This can lead to an overestimation of the LLS and therefore an overestimation of the predicted dose to the lungs. Planar scatter correction was able to reduce the apparent lung shunt to values comparable to those measured with ^99m^Tc. Other studies have also found an overestimation of the LLS using ^166^Ho planar images [3, 15]. In contrast to the study by Stella et al., we found smaller apparent lung shunt values, which could be due to the use of different phantoms. Measurements of the NEMA IQ phantom with activity only in the spheres suggest that small extrahepatic depositions can be detected with ^99m^Tc as well as ^166^Ho. The NEMA IQ phantom images with 8:1 and 4:1 sphere-to-background activity concentration ratios showed worse detectability of small spheres with ^166^Ho compared to ^99m^Tc. This means that the activity accumulation in small liver metastases may not be detectable in SPECT/CT images with ^166^Ho and could not be considered for treatment planning and dosimetry. For both isotopes, the resolution and the large partial volume effect can have an impact on predictive voxel-based dosimetry. Predicted tumor absorbed doses may be underestimated in small lesions due to underestimation of activity in small volumes. This underestimation of activity is less pronounced for ^99m^Tc than for ^166^Ho due to the better resolution and contrast recovery. All results in this study are based on phantom measurements which represent ideal measuring conditions, meaning homogenous activity distribution and spherical activity spots. In vivo imaging conditions such as irregularly shaped lesions and inhomogeneous distribution may have additional influences on scintigraphic imaging that could not be observed in this study. Furthermore, the influence of the reconstruction algorithm and the reconstruction parameters were not investigated. Monte Carlo-based reconstruction algorithms, including simulation of photon scattering and collimator-detector response, can improve image quality, but are usually not available for clinical use [11].

Personalized dosimetry, taking into account the activity distribution in the tumor and parenchyma, is becoming increasingly important, as a significant dose-response relationship has been confirmed for radioembolization [24–27]. Prerequisites for individualized dosimetry are a good agreement between the microsphere distribution in diagnostics and therapy, and additionally an accurate imaging of the microsphere distribution. In addition to the type of particles used, intrahepatic activity distribution is significantly influenced by catheter position, regional vasculature, and hemodynamics. The ^166^Ho scout dose provides a good prediction of particle distribution because the same microspheres are used for diagnostics and therapy [5]. However, the poor imaging characteristics of ^166^Ho can have a negative impact on individualized dosimetry. Therefore, it has to be analyzed whether the use of ^99m^Tc-labeled PLLA microspheres allows a superior imaging of the particle distribution and therefore a more accurate predictive dosimetry. In future studies the labeling of PLLA microspheres with ^99m^Tc and a comparison of the in vivo distribution of ^99m^Tc-PLLA and ^166^Ho-PLLA should be investigated.

## Conclusion

In summary, our measurements revealed that planar and SPECT imaging is better with ^99m^Tc compared to ^166^Ho regarding spatial resolution, contrast recovery, image noise, and lesion detectability. Therefore, ^99m^Tc is the more appropriate isotope for pre-treatment imaging in SIRT. The worse imaging characteristics of ^166^Ho can have a negative influence on individualized treatment planning. However, ^166^Ho scout dose provides a good prediction of particle distribution since the same microspheres are used for therapy. Further investigations regarding ^99m^Tc-labeled PLLA microspheres, combining the better scintigraphic imaging properties of ^99m^Tc with the advantages of the PLLA microspheres, have to be performed.

## Data Availability

The datasets used and/or analyzed during the current study are available from the corresponding author on reasonable request.

## Abbreviations

SIRT: Selective Internal Radiation Therapy
LLS: Liver lung shunt
PLLA: Poly–L–lactic–acid
FOV: Field of view
LEHR: Low–energy high–resolution
MELP: Medium–energy low–penetration
IQ: Image quality
ROI: Region of interest
OSEM: Ordered Subset Expectation Maximization
VOI: Volume of Interest
CRC: Contrast Recovery Coefficient
CNR: Contrast–to–noise ratio
FWHM: Full width at half maximum
cps: Counts per second
